# Cardiopulmonary Manifestations of Ankylosing Spondylitis

**DOI:** 10.1155/2011/728471

**Published:** 2011-04-12

**Authors:** Mahnaz Momeni, Nora Taylor, Mahsa Tehrani

**Affiliations:** ^1^Rheumatology Division, The George Washington University Medical Center, 2150 Pennsylvania Avenue, 3-416 NW, Washington, DC 20037, USA; ^2^Internal Medicine Division, The George Washington University Medical Center, Washington, DC 20052, USA

## Abstract

Ankylosing spondylitis is a chronic inflammatory condition that usually affects young men. Cardiac dysfunction and pulmonary disease are well-known and commonly reported extra-articular manifestation, associated with ankylosing spondylitis (AS). AS has also been reported to be specifically associated with aortitis, aortic valve diseases, conduction disturbances, cardiomyopathy and ischemic heart disease. The pulmonary manifestations of the disease include fibrosis of the upper lobes, interstitial lung disease, ventilatory impairment due to chest wall restriction, sleep apnea, and spontaneous pneumothorax. They are many reports detailing pathophysiology, hypothesized mechanisms leading to these derangements, and estimated prevalence of such findings in the AS populations. At this time, there are no clear guidelines regarding a stepwise approach to screen these patients for cardiovascular and pulmonary complications.

## 1. Cardiac Manifestations of Ankylosing Spondylitis

**Introduction**

Ankylosing spondylitis is a chronic and inflammatory condition, affecting the spine, sacroiliac, and peripheral joints. This entity most often affects young men and may lead to spinal vertebral fusion. Human leukocyte antigen (HLA)-B27 is present in the majority of patients with AS and is reported to contribute to the pathophysiologic manifestations of this condition [1].

It has been estimated that cardiac manifestations in patients with AS are found in 2–10% of patients. It was initially in the 1930s when aortitis found in a group of patients with spondylitis. It is widely accepted today that not only is aortic pathology linked to AS, but there is also risk for conduction defects, valvular regurgitation, and cardiomyopathy, associated with this entity [1]. This is especially important, given that in many patients, cardiac changes may begin prior to the onset of clinical symptoms [2, 3].

### 1.1. Valvular Disease

The presence of aortic root and valve disease in ankylosing spondylitis is related to the duration of the underlying disease. Aortic disease and aortic regurgitation may, however, predate the onset of any joint symptoms, and the presence of ankylosing spondylitis as an underlying cause may not be initially appreciated [1].

One of the first pathophysiologic descriptions of valvular disease in AS was put forward by Bulkley and Roberts, who studied autopsy findings in eight patients with AS. They noted aortic root dilatation along with fibrous proliferation along the intima [4]. Further examination demonstrated a cellular inflammatory process coupled with platelet aggregation leading to endarteritis around the aortic root and valve. This in turn stimulates fibroblast hyperactivity leading to tissue thickening involving aortic annulus, cusps, aorto-mitral junction along with the conduction system [5]. The thickening of the valvular cusps, dilatation of the aortic root, and abnormal cusp displacement via the thickened subaortic tissue all lead to aortic regurgitation as seen in many patients with AS [1].

Roldan et al. studied the root and valves in AS patients (*n* = 44) using transthoracic echocardiography (TEE) and found aortic root and valve disease in (82%) as compared with control (27%). Other irregularities were aortic root thickening, increased root stiffness and dilatation. Except for the duration of AKS, aortic root disease and valve disease were unrelated to the activity, severity or therapy of AKS. During followup of 25 patients over 39 months in this study, in up to 24% new aortic root or valve abnormalities developed, in 12% existing valve regurgitation worsened significantly and in 20% abnormalities resolved. Twenty percent of patients developed heart failure, underwent valve replacement [5]. Aortic regurgitation is most commonly seen in patients with AS; however, mitral regurgitation is also known to occur, although less common [1]. The proposed mechanism is basically a continuation of the fibrosis of the subaortic tissues which can progress to reach the mitral valve leaflet. Thereby, a stiffening and hence reduction of the mitral valvular mobility occurs, eventually leading to mitral regurgitation. Another potential mechanism for mitral regurgitation may be as a result of severe aortic regurgitation, leading to a hypertrophied left ventricle and hence a distortion of the mitral valve apparatus [1].

### 1.2. Conduction and Rhythm Abnormalities

Conduction abnormalities are amongst the most commonly observed cardiac manifestations of patients with AS that usually predate other cardiac manifestations such as valvular insufficiency [6]. In a study by Dik et al. they found a statistically significant higher prevalence of AS patients with first-degree AV block as well as an association of these blocks with the duration of disease activity [7]. There have been two prevalent theories about the etiology of conduction disturbances in patients with AS: inflammation in the intraventricular septum leading to damage versus anomalies in the AV nodal artery leading to AV node dysfunction [7]. Certainly, the above processes may play hand in hand, given that both AV blocks along with atrial and ventricular extra-systoles have been reported [7, 8]. Interestingly, in the observational study by Dik et al. they also report an increased finding of conduction disturbances in patients who are HLA-B27 positive, even in patients without rheumatologic manifestations of AS [7]. 

Toussirot et al. found factors which highlight autonomic nervous system derangements, in patients with AS [9]. For example, there was a finding of a small but statistically significant elevation in heart rates. This correlated with disease severity, as measured by ESR and CRP levels [9]. Furthermore, an inability to maintain steady arterial blood pressures with changes in position (i.e., supine to erect), was found. Based on their findings, the group postulated that these autonomic derangements may lead to conduction defects and arrhythmias, thereby negatively impacting prognosis. They further extrapolated that these subtle autonomic findings may be evident prior to more concrete EKG findings, hence a clinical practice implication [10]. Contradictory to this report is a paper two years later, by Yildirir et al. refuting autonomic dysfunction in the AS population [10]. This group utilized heart rate variability as a measure of autonomic control and found no statistically significant difference between the AS versus the control group. An acknowledged limitation was the young population in this latter study, along with the fact that all patients were under significant treatment with antiinflammatory agents, hence hypothetically halting the advancement of autonomic dysfunction [10]. 

Since most of the studies points towards early detection and treatment of rheumatologic cardiac manifestations, then early testing of these patients becomes important [8]. There are multiple modalities listed in the literature to aid in the evaluation of cardiac manifestations of rheumatic disease, such as utilization of 24-hour holter monitoring and echocardiography [8]. In a study by Yildirir et al. they demonstrate that a simple way to evaluate for the presence of or propensity for developing arrhythmias is via measuring QT dispersion. Yildirir et al. demonstrated that calculation of the QT dispersion on a standard EKG is cheap, quick and reveals valuable information regarding patient susceptibility to arrhythmias. This approach is way more cost effective than sending all patients off for holter monitoring amongst other tests, and can easily be instituted in-office, whereby only patients with increased arrhythmia risk can be referred for further workup [8].

### 1.3. Myocardial Disease

It is widely demonstrated that the myocardial dysfunction evident in patients with AS is that of the diastolic variety, as opposed to systolic. For example, in a study by Yildirir et al. they found significant diastolic dysfunction in AS patients, particularly an abnormal relaxation pattern [2]. This was demonstrated via echocardiography, by the statistically significant lower E-wave velocity (early diastolic filling), higher A-wave velocity (late diastolic filling), and overall low E/A ratio, which becomes evident in patients with diastolic dysfunction [2]. Similarly, Gould et al. evaluated the peak filling throughout exercise, along with the time to achieve peak filling during strenuous activity via radionucleotide angiography. Both of these measures were considerably decreased in the patients with AS versus the normal volunteers [11]. Hence, these findings also support an overall decrease in compliance, and thus a reduction in diastolic function. 

Coronary flow reserve (CFR) is a measurement, which evaluates the function of the coronaries in terms of their microvascular circulation. This measure has been touted by some experts as an important tool, holding prognostic significance in the evolution of atherosclerosis in patients [3]. Caliskan et al. recently used transthoracic Doppler echocardiography (TTDE) to determine the CFR in AS patients. Their findings were consistent with a significant decrease in CFR in AS patients. Interestingly, the levels of CRP and TNF-alpha correlated with the decline in CFR in these patients [3]. 

Another study looked at an actual outcome, being myocardial infarction, in patients with AS. They found a 2-3 fold increase in the rate of infarctions in the AS population which they studied. Of note, amongst AS patients with and without a myocardial infarct history, there was no difference in the use and type of antirheumatic therapy [12]. This is a significant observation; however, difficult to interpret as the sample size is very limited (17 patients). A study with multiple antirheumatic treatment arms would be ideal for this type of analysis. 

There has been research demonstrating that markers of inflammation (i.e., CRP, IL-6) can reach a high enough level to start disturbing lipid metabolism and diminishing insulin activity, hence causing insulin resistance and dyslipidemia, leading to increased overall trend for atherogenecity [13]. In a study by Divecha et al. they found a significant elevation in IL-6 and CRP levels in patients with AS, over that of healthy subjects. This is significant, as it has been reported that even a subclinical but chronic elevation in the levels of these markers accelerate risk of coronary heart disease, in patients without autoimmune illnesses [13]. Furthermore given that the clinical use of statins in a rheumatoid arthritis trial not only helped with lipids but also led to reduced IL-6 levels, so it is important to assess the role of these pharmacologic agents in patients with AS and other spondyloarthritides [13–15].

## 2. Pulmonary Manifestations of Ankylosing Spondylitis

**Introduction**

Pulmonary involvement in ankylosing spondylitis is an extra-articular manifestation of the disease that was first described in 1941 in an article reviewing twenty cases of ankylosing spondylitis [16] when two cases were noted to have “healed apical tuberculosis”. The pulmonary manifestations of the disease include fibrosis of the upper lobes, interstitial lung disease, and ventilatory impairment due to chest wall restriction, sleep apnea, and spontaneous pneumothorax. The incidence of lung involvement in ankylosing spondylitis has changed with the development of high-resolution computed tomography (HRCT). Although improved techniques in visualization of the lung have allowed identification of more abnormalities of the lung parenchyma associated with ankylosing spondylitis there is little known about the natural history of such abnormalities and the potential for therapies used in treatment of ankylosing spondylitis to halt the progression of the lung disease.

### 2.1. High-Resolution Computed Tomography

The introduction of HRCT in pulmonary evaluation of ankylosing spondylitis patients has shown that lung parenchymal changes occur earlier and are more extensive than once believed. In an evaluation of 26 patients with ankylosing spondylitis for less than 5 years by Baser et al. HRCT changes were seen in 13/25 (50%) of patients [17]. Upper lobe fibrosis was identified in 1.3% of 2080 cases in a retrospective review in 1977 but recent studies using HRCT have shown a more extensive involvement of the lung [18]. The use of HRCT for evaluation of whole lung involvement was first utilized by Casserly et al. in 1997 revealing lung involvement in 19/26 (70%) ankylosing spondylitis patients meeting the New York criteria for ankylosing spondylitis. This uncontrolled study using HRCT to examining the lung parenchyma saw a far larger number of abnormalities on HRCT as compared with only 4/26 (15.3%) abnormal plain X-rays in the same patients. Lung parenchymal abnormalities observed included interstitial lung disease, emphysema, apical fibrosis, mycetoma, and nonsepcific intestinal lung disease [19]. Sampaio-Barros et al. evaluated pulmonary involvement of ankylosing spondylitis in 52 patients with no lung complaints and found that 40% had abnormalities by HRCT compared with 8% by traditional chest X-ray [20]. A similar finding was seen in a study by Turetschek et al. in which 25 nonsmoking ankylosing spondylitis patients underwent pulmonary function testing, chest X-ray, and thin section CT with 15/21 (71%) of patients found to have abnormalities on thin-section CT [21].

### 2.2. Apical Fibrosis and Interstitial Lung Disease

Upper lobe fibrosis is a long recognized lung abnormality associated with ankylosing spondylitis. The incidence of apical fibrosis in ankylosing spondylitis is low with estimates ranging from 1.3–30% and has an association with longer disease duration [22]. Apical fibrosis often appears greater than five years after the start of the arthritic symptoms associated with the disease [23]. The fibrosis can be unilateral or bilateral in nature and is can be followed by cystic changes to the lung [24]. The cause of the fibrosis is unknown but recurrent aspiration leading to aspiration pneumonitis from defective ventilation, alterations in apical mechanical stress from a rigid thoracic spine, and recurrent impaired cough secondary to alterations in respiratory mechanics have been proposed [25, 26]. The abnormal lung parenchyma is a fertile bed for superinfection with a variety of organisms including atypical *Mycobacterium*, *Mycobacterium tuberculosis*, *Aspergillus*, and *Metschnikowia pulcherima* [27–29]. The distribution and appearance of the upper lobe abnormalities in ankylosing spondylitis patients led many physicians in the past to incorrectly diagnose these patients with *Mycobacterium tuberculosis.* However, Ho et al. in a study in Taiwan illustrated how differentiation between the two often requires further diagnostic evaluation and a high index of suspicion for infection when review of medical records of 2136 ankylosing spondylitis patients in Taiwan revealed 63/2136 (2.9%) with apical fibrosis and chronic infection from *Mycobacterium Tuberculosis *found in 41/63 (65%) of those patients [30].

Interstitial lung disease (ILD), beyond apical fibrosis, is now a recognized feature of pulmonary involvement in ankylosing spondylitis as a result of improved visualization of the lung parenchyma with HRCT [19]. Pathologic diagnosis of ILD in the setting of ankylosing spondyltitis has rarely been described due to a relative paucity of autopsy studies and the cause of the ILD in this setting is still unclear. Case reports and small series to date have shown evidence of chronic inflammatory cell infiltrates and prominent interstitial fibrosis with elastic degeneration of collagen and hyaline by needle biopsy and lobectomy examinations [31]. One case study in 1971 described a patient with longstanding ankylosing spondylitis admitted to the hospital with pancytopenia and diffuse bilateral interstitial and alveolar infiltrates involving the middle and upper lung fields [32]. Percutaneous lung biopsy was performed and revealed interstitial pneumonitis and fibrinous alveolitis. 

Multiple studies have shown the association between ILD and ankylosing spondylitis [17, 19, 33]. Baser et al. was the first to show that these parenchymal changes can occur early in the course of the disease, less than 5 years after the start of ankylosing spondylitis symptoms, with parenchymal abnormalities in 13/26 (50%) of all patients, 8/21 (38.1%) of asymptomatic patients, and in 5/5 (100%) of symptomatic patients. The parenchymal abnormalities were broad including emphysema, bronchiectasis, ground glass opacities, septal thickening, parenchymal micronodules, pleural thickening, parenchymal bands, and apical fibrosis with emphysema being the most common abnormality seen in 9/21 (34.6%) of patients. Similar findings were seen by Souza et al. with 11/17 (65%) of patients having evidence of ILD on HRCT [33]. In an observational study of HRCT in ankylosing spondylitis, Casserly et al. [19] found interstitial lung disease in 4/26 (16%) of ankylosing spondylitis patients. Simultaneous evaluation with chest X-ray did not reveal any abnormalities in any of the 4 patients found to have ILD on HRCT, however, all 4 patients had abnormal pulmonary function testing.

### 2.3. Chest Wall Restriction and Ventilatory Abnormalities

Restricted chest wall motion can lead to restricted pulmonary function. Dorsal kyphosis from involvement of the thoracic spine, costoverterbral, sternoclavicular, and sternomanubrial joints leads to impairment of chest wall expansion with breathing. Disease activity was not associated with PFT abnormalities in one study evaluating 36 ankylosing spondylitis patients compared to 34 controls who underwent pulmonary function testing, smoking and quality of life questionnaires [34]. The opposite was seen in an alternate study by Maghraoui et al. of 55 ankylosing spondylitis patients with a statistically significant correlation between disease activity and PFT abnormalities [35]. The predominant abnormality on pulmonary function testing is a restrictive pattern and has been seen in numerous studies [31]. 

### 2.4. Spontaneous Pneumothorax

Spontaneous pneumothorax is a very rare complication of ankylosing spondylitis, with one retrospective review in Taiwan revealing a total of 3/1028 cases for an incidence of 0.29% [36]. The three cases were all seen in the setting of patients with apical lung fibrotic changes confirmed by X-ray. Apical lung abnormalities were seen in 22 patients total (22/1028, 2.1%), with spontaneous pneumothorax in 3 (3/22, 13.6%) leading the authors to propose the primary risk factor for spontaneous pneumothorax in ankylosing spondylitis patients to be apical fibrosis. All three of the individuals who had a spontaneous pneumothorax related to their disease happened to be smokers. The contribution of smoking to the risk of spontaneous pneumothorax in ankylosing spondylitis patients is unclear given the small numbers of spontaneous pneumothorax overall. Mean duration of time from diagnosis of ankylosing spondylitis to timing of spontaneous pneumothorax was 13.0 ± 6.2 years. Recurrence of spontaneous pneumothorax occurred in 2 out of 3 cases with the third case undergoing talc pleurodesis at the first occurrence leading the authors to conclude in their paper with a recommendation that a prophylactic procedure to prevent recurrence be considered at the first event. This was echoed in a case report published by Kaneda et al. discussing a case of recurrent bilateral repeat pneumothorax in a 53-year-old male with ankylosing spondylitis who ultimately required bilateral chemical pleurodesis [37].

### 2.5. Sleep Apnea

An increased incidence of sleep apnea in patients with ankylosing spondylitis has been described in several studies [38]. Fatigue is a common symptom in ankylosing spondylitis and is a question on the Bath Ankylosing Spondylitis Disease Activity Index (BASDAI) used to determine the severity of an individual patients' disease [39]. Obstructive sleep apnea was found in 12% of patients in a small study by Erb et al. of 17 patients with ankylosing spondylitis. This percentage was significantly higher than the 1–4% seen in the general population. Possible mechanisms for the cause of obstructive sleep apnea in ankylosing spondylitis put forward by Erb et al. include restriction of the oropharyngeal airway by compression from cervical spine involvement or temporomandibular involvement, restrictive pulmonary disease, or cervical spine disease causing compression of the respiratory centers found in the medulla [40]. In an attempt to further assess the prevalence of obstructive sleep apnea in ankylosing spondylits, Solak et al. [38] followed with a study of 31 ankylosing spondylitis patient and subjected all patients to a polysomnography evaluation and pulmonary function testing. Results revealed 7/31 (22.6%) found to have obstructive sleep apnea with the mean age of those suffering from sleep apnea (43.4 ± 5.7) being statistically higher than those without sleep apnea (33.2 ± 10.6). Pulmonary function testing of the same group revealed a restrictive pattern in 12 (53.3%) of patients with no increased incidence of pulmonary function abnormalities in the group with obstructive sleep apnea as compared to those without sleep apnea. An obstructive pattern on pulmonary function testing was not observed in any patients. 

Given the increased incidence of obstructive sleep apnea in ankylosing spondylitis, formal sleep evaluation should be considered in patients complaining of severe fatigue. Treatment of obstructive sleep apnea in ankylosing spondylitis is the same as for patients without ankylosing spondylitis and includes CPAP (continuous positive airway pressure), smoking cessation, and, if needed, weight loss to achieve a normal body mass index.

Recent studies on sleep disturbance in ankylosing spondylitis look at the effect of golimumab and adalimumab in the reduction of sleep disturbance in patients suffering from ankylosing spondylitis [41, 42]. Neither study addressed the issue of sleep apnea specifically but instead looked at validated measures of sleep disturbance and improvement of these measures with the use of golimumab and adalimumab. Deodhar et al. looked at sleep disturbance in patients treated with golimumab 50 or 100 mg as compared with placebo using the Jenkins Sleep Evaluation Questionnaire (JSEQ). Patients on both doses of golimumab had a significant greater improvement of their JSEQ compared to placebo from baseline to week 12 and week 24 [41]. Rudwaleit et al. performed a similar subanalysis of the RHAPSODY (Review of Safety and Effectiveness with Adalimumab in Patients with Active Ankylosing Spondylitis) to assess the effect of adalimumab on sleep after 12 weeks of treatment using the Medical Outcomes Sleep Study Scale (MOS-SS) and showed that at week 12, adalimumab improved sleep in all of the MOS-SS domains. Improvement was similar in both patients with and without radiographically advanced ankylosing spondylitis [42]. While both of these studies indicate ankylosing spondylitis improves patients' assessment of sleep disturbance, interesting future studies of antiTNF*α* agents in ankylosing spondylitis could include assessment of sleep apnea in ankylosing spondylitis and whether it can be reversed with antiTNF*α* therapy alone.

## 3. Conclusion

Knowledge on the various pulmonary and cardiac manifestations of ankylosing spondylitis has expanded throughout the years with further study and improved imaging techniques. The are many reports detailing pathophysiology, hypothesized mechanisms leading to these derangements, and estimated prevalence of such findings in the AS populations. At this time, there are no clear guidelines regarding a step-wise approach to screen these patients for cardiovascular or pulmonary complications. While some investigators clearly hint at the significance of detecting cardiopulmonary disease as early as possible via clinical exams, lab tests, EKGs, echocardiography, PFT, CT scan, there are some that remain inconclusive in their recommendations. 

A large prospective cohort study might be able to find out if early detection of cardiopulmonary disease can change patient's prognosis and provide more concrete data by which to recommend routine cardiac and pulmonary testing in ankylosing spondylitis.

 Similarly, Antitumor necrosis factor (TNF) *α* therapy has now been used in the treatment of patients with ankylosing spondylitis with good success in terms of improving functionality, pain scores, and sleep disturbance [41–44]. Whether antiTNF*α* can halt or prevent the pulmonary or cardiac manifestations of the disease will require a large prospective trial in the future and must weigh the unknown risks of prolonged treatment with antiTNF*α* agents against treatment of ankylosing spondylitis and its extra-articular manifestations.
